# Role of Gut Microbiota in the Pharmacological Effects of Natural Products

**DOI:** 10.1155/2019/2682748

**Published:** 2019-04-15

**Authors:** Chuyue Pan, Qinglong Guo, Na Lu

**Affiliations:** State Key Laboratory of Natural Medicines, Jiangsu Key Laboratory of Carcinogenesis and Intervention, Department of Basic Medicine, School of Basic Medicine and Clinical Pharmacy, China Pharmaceutical University, 24 Tongjiaxiang, Nanjing 210009, China

## Abstract

Increasing evidence has demonstrated that natural products derived from traditional Chinese medicine, such as ginseng, berberine, and curcumin, possess a wide variety of biological activities on gut microbiota, which may cause changes in the composition of intestinal microbiota, microbial metabolites, intestinal tight junction structure, and mucosal immunology. These changes will eventually result in the exertion of the pharmacological effects by treatment with these natural products. In this review, we will discuss how gut microbiota is influenced by commonly used natural products. Furthermore, our findings are expected to provide novel insight into how these untargeted natural products function via gut microbiota.

## 1. Introduction

The relationship between gut microbiota and human diseases has been a major topic of interest for many studies. Increasing evidence has suggested that gut microbiota plays an important role in diseases, including obesity [1], type 2 diabetes mellitus (T2DM), cardiovascular diseases [2], and cancer [3, 4]. In many studies, bacteria-deficient mice have been reported to consume more calories than normal mice [5], and it was more difficult for these mice to become obese [6]. Studies in germ-free mice have indicated that gut microbiota was associated with weight gain, lipid synthesis, and fat storage. In addition, high-fat diet- (HFD-) induced obesity is usually accompanied with diabetes [7], cardiovascular diseases, and liver diseases such as nonalcoholic fatty liver disease (NAFLD). Besides, gut microbiota, which can be part of the tumor microenvironment, communicates with tumor cells, and some immune cells [8], and may be the key factor in the process of HFD-induced cancer progression [9].

In recent years, interest in the pharmacological activities of natural products has significantly increased.* In vitro* studies and clinical data have demonstrated positive effects of natural products on many diseases [10, 11]. The aim of this study was to discuss the effects of natural products on the composition of gut microbiota, metabolites, intestinal tight junction proteins, and mucosal immunity and to provide novel insight into future therapies.

## 2. Effect of Natural Products on Composition of Gut Microbiota

The gut microbiota is hypothesized to play a critical role in metabolic diseases, such as obesity and T2DM. An increasing number of studies have shown that natural products exert antiobesity activities by modulating the composition of gut microbiota. For example, MDG-1, a water-soluble polysaccharide extracted from the root of* Ophiopogon japonicus* Ker Gawl has been reported to regulate body metabolism, including weight loss, antiobesity, and antidiabetes. Shi et al. [12] demonstrated that, in HFD-induced obese mice that were treated with MDG-1 at a high dose of 300 mg/kg for 12 weeks, the ratio of Firmicutes/Bacteroidetes (F/B) decreased to normal levels. In addition, it was found that, in a HFD-induced diabetic mouse model, MDG-1 decreased the number of pathogenic bacteria (*Escherichia coli* and* Streptococcus*) [13]. Green tea, commonly consumed in Asia, has also been reported to have an antiobesity activity [14], and the alteration of gut microbiota composition has been presumed as one of its mechanisms of action. When sundried green tea was fermented, it restored the increased Bacteroides/Prevotella (B/P) ratio [15] and significantly decreased the F/B ratio in HFD mice after 8 weeks of treatment. Epigallocatechin gallate (EGCG), the main type of catechin in green tea, could inhibit the formation of rat abdominal adipose tissue after a 4-week treatment regimen. In their study, it was revealed that EGCG-treated rats showed a dramatic decrease of* Clostridium *spp. and an increase of Bacteroides in feces [16]. Moreover, Chang et al. [17] reported that a water extract of* Ganoderma lucidum* (Curtis) P. Karst. (WEGL) prevented weight gain and fat accumulation in HFD-induced obese mice. Furthermore, endotoxemia and insulin resistance were found to be improved by WEGL for the modification of gut dysbiosis. The F/B ratio and levels of endotoxin-bearing Proteobacteria were also restored to normal levels. However, several bacteria increased (*Parabacteroides goldsteinii, Bacteroides *spp*., Anaerotruncus colihominis, Roseburia hominis, and Clostridium*), which negatively correlated with obesity. The authors suggested that WEGL or polysaccharides could be used as prebiotic agents for the treatment of obesity and modulating obesity-related metabolic disorders [17].

*Lonicerae japonicae flos* is famous for its anti-inflammatory activity and has widely been used in Asia for years. In animal studies using HFD-fed animals, administration of unfermented* Flos Lonicera* (UFL) or fermented* Flos Lonicera* (FFL) significantly reduced body weight (BW) and adipose tissue weight and decreased lipid accumulation in the liver with ameliorated serum total cholesterol, HDL, and triglyceride levels. As suggested by the authors, alterations in the relative abundance of* Lactobacillus *spp.,* Bifidobacterium *spp*.,* and B/F ratio in the intestinal tract were supposed to be one of the mechanisms of UFL or FFL [18]. In addition, the amount of* Bifidobacterium *spp. in the cecal pool of HFD-induced mice was increased by pomegranate peel extract (PPE), which is known for its beneficial effects, including anti-inflammatory and antimicrobial activities [19].

Berberine, the main active ingredient of Chinese herb Coptis chinensis, is known as an antidiabetes drug and can regulate blood glucose [20, 21]. Berberine has been shown to be beneficial for HFD-induced insulin resistance, as it improves insulin sensitivity and reduces the homeostasis assessment of the insulin resistance (HOMA-IR) value. As reported by Sun* et al*., administration of berberine reduced the ratio of F/B and partly recovered the composition of gut microbiota changed by HFD feeding. Furthermore, it also showed that both the diversity and richness of gut microbiota were significantly decreased after berberine administration [22]. These findings were supported by Zhang* et al*., who showed that the partial least squares (PLS) regression model predicted the relationship between the changes in composition of gut microbiota and host phenotype [23].

In addition to animal studies, several studies of natural products have been performed on human gut microbiota. Xu* et al.* reported that Gegen Qinlian Decoction (GQD), a traditional Chinese herbal formula, reshaped the gut microbiota in a clinical study in which 187 T2D patients were enrolled. The data showed that the symptoms of T2D, such as fasting blood glucose levels and hemoglobin A1c (HbA1c) levels, were ameliorated in GQD-treated patients, with increased amounts of beneficial bacteria, including* Faecalibacterium*,* Gemmiger*,* Bifidobacterium, *and* Escherichia* [11]. Another clinical study involving ten obese Korean women was conducted to investigate the antiobesity activity of the water extract of Ephedra sinica Stapf, which revealed that, in seven of the ten obese women, BW and body mass index (BMI) were decreased after administration of this herb. Interestingly, the antiobesity effect of ginseng varied when the composition of gut microbiota was altered. The abundance of* Subdoligranulum*,* Oscillibacter*, and* Akkermansia* in the gut associated with changes in BW and BMI, whereas* Lactobacillus* was linked to body fat percentage [10].

Natural products also play an important role in the improvement of gastrointestinal tract function. Several Chinese medicine products such as Red Ginseng and Semen Coicis were found to relieve the symptoms of ulcerative colitis (UC) [24, 25]. After Red Ginseng and Semen Coicis treatment in rats, the structure of gut microbiota was altered, which may be beneficial for promoting the growth of probiotics, such as* Bifidobacterium* and* Lactobacillus*, and for inhibiting the growth of pathogenic bacteria [25]. In a double-blind, randomized clinical trial containing 54 patients, it was revealed that the combination of herbal medicine (*Gwakhyangjeonggisan*, GJS) and probiotics (Duolac7S, DUO) alleviated the symptoms of diarrhea-predominant irritable bowel syndrome (D-IBS) by changing the composition of gut microbiota. Beneficial intestinal microbe counts, including* Bifidobacterium brevis*,* Bifidobacterium lactis*,* Streptococcus thermophilus*,* Lactobacillus rhamnosus*,* Lactobacillus plantarum, and Lactobacillus acidophilus,* were synergistically enhanced by GJS combined with DUO, suggesting that a combined treatment of herbal medicine and probiotics might provide a promising implication for clinical treatment of D-IBS [26, 27].

## 3. Effect of Natural Products on Metabolites of Gut Microbiota

In the human intestine, microbes are vital contributors to the host metabolism, considering that numerous important components, such as vitamin K, folate, indoles, gamma amino butyric acid, and short-chained fatty acids (SCFAs), are produced by microbiota [28–30]. In general, these metabolites are involved in many physiological and pathophysiological processes, which may be related to several diseases, including cardiovascular diseases [29], allergic reactions [30], T2DM [31], and various types of cancers [8, 32]. By changing the bacterial structure, natural products may regulate the metabolism of the microbiome, Zhao* et al*. found that the abundance of genes encoding SCFA production and the fecal butyric acid concentration were notably increased by a high fiber diet from traditional Chinese medicinal food plants, and several T2DM parameters were found to be improved after treatment, and they identified 15 positive responders of SCFA which played an important role in maintaining intestinal homeostasis [31]. When total saponins and polysaccharides (active constituents of* P. kingianum*) were administrated to diabetic rats, the abundance of the content of certain bacterial taxa and fecal SCFAs in rats was upregulated, while the lipopolysaccharide (LPS) concentration was downregulated [33]. These results suggested that natural products might be the treat levels of protective metabolites and that detrimental metabolites can contribute to metabolic diseases. Chen* et al*. reported that sulfate-reducing bacteria were significantly decreased after* Gynostemma pentaphyllum* (GpS) treatment in* Apc*^*Min /+*^ mice. As a result, the level of harmful molecular hydrogen sulfide produced by sulfate-reducing bacteria was also reduced [34].

Gut microbiota can also impact the metabolism of natural products and therefore influence their treatment effects. It has been demonstrated that commensal bacteria regulate countless genes involved in drug metabolism and hepatic expression of drug-metabolizing enzymes [35–37]. Therefore, the microbial activity in metabolic phenotype development is important, especially for pharmacokinetics [35], because treatment effects of components of traditional Chinese herbs may be enhanced by biotransformation. For instance, many glycoside compounds are hydrolyzed by gut flora when administered orally, and thereby, the solubility is improved to allow for easy absorption. Ginsensoide Rb1 [37] and aglycone of baicalin [38] are biotransformed to a more active form, aglycone. After transformation, they harbor a much better bioavailability. On the contrary, the activity of several molecules can be transformed to an inactive form and reduce their bioactivities. Ru* et al*. found that berberine is metabolized to dihydroberberine (dhBBR), which is easier to absorb but has less active properties, although in the blood it can be oxidized to berberine [39]. Several herbs are toxic and their harmful metabolites can also be more or less toxic [40, 41]. Human intestinal microbiota can metabolize aconitine, an alkaloid that can induce systemic acute toxicity, to be less harmful to the body through acetylation and esterification [40]. However, aristolochic acid in* Radix Aristolochiae Fangchi* can cause acute renal injury, and long term administration can increase the risk of cancer. Its main microbial metabolite aristolochic acid I, the most toxic component of the Aristolochia herbs, is particularly cytotoxic in the kidney [42].

From another point of view, gut microbiota has played an important role on the pharmacokinetics and pharmacodynamics of natural products. The loss of gut microbiota or dysbiosis may reduce the efficacy of traditional Chinese medicine. Liu et* al.* reported that rats treated with broad-spectrum antibiotics show a different pharmacological response to Shaoyao-Gancao decoction (SGD) when compared with control mice. The antibiotics inhibited the absorption of SGD and reduced biotransformation of SGD in the colon. Two constituents of SGD have significantly reduced AUC_0-24h_ after antibiotics treatment, but the half-lives (T_1/2_) and mean retention times (MRT) did not remarkably change [43]. It seems that gut microbiota mainly affects the blood concentration and AUC of herb medicines. Furthermore, Shen* et al.* used 5% dextran sulfate sodium (DSS) to disturb gut microbial homeostasis and observed a quicker absorption (less T_max_) and a lower max concentration (C_max_) of ginsenoside Rb1 in DSS-treated mice. Once given drugs to restore the balance, these changes recovered, which indicated the importance of the intestinal flora in drug metabolism [44]. Thus, it is of importance to consider the influence of gut microbiota in clinical medicine. Digoxin is an example of a drug that decreases drug efficacy when it metabolized by microbiota. It has been reported that gut microbiota can degrade digoxin to its inactive form, such as dihydrodigoxin and dihydrodigoxigenin [45], which hardly bind Na^+^-K^+^-ATPase of cardiac cells [46]. Conversely, some new effects can be functioned during the interaction between drugs and gut microbiota. In a study by Ru et al., the mechanism of antihyperlipidemia function and pharmacokinetics in beagle dogs was determined. Different from effects such as anti-inflammatory and antidiabetes activities, berberine induced the production of butyrate by upregulating butyrate-producing microbiota [47]. All studies involving novel effects or mechanism may have direct applications to clinical medicines.

In conclusion, natural products can affect the metabolism of gut microbes, and the microbiome can change the metabolic process of natural components.

## 4. Effect of Natural Products on Improving Gut Tight Junction through Regulating Intestinal Microbes

In intestinal epithelial cells, tight junctions usually function as a barrier to defense against bacterial endotoxin. In addition, LPS-induced disruption of tight junctions can lead to dysregulation of intestinal epithelial cells, as well as of the immune system [48]. A HFD could increase LPS release induced by gut microbiota, thereby impairing the expression of tight junction proteins leading to the increase of intestinal permeability [49]. Furthermore, HFD elevated the LPS concentration in plasma, and subsequently increased the secretion of adipokines. In a recent study, it was shown that* Lactobacilli* positively associated with human BMI and blood glucose values [50]. In addition, it was reported that* Lactobacilli* could reduce the LPS level, decrease blood glucose levels, and inflammation in HFD mice. Besides,* Lactobacilli sakei *OK67 could restore the expression of colonic tight junction protein expression and ameliorate HFD-induced hyperglycemia [51].

Moreover, in previous studies, it was demonstrated that nanoparticle loaded with berberine could protect tight junction against inflammation induced by LPS. And berberine-loaded nanoparticles turned out to be useful in the restoration of tight junctions in intestinal epithelial cells (IEC) [52]. Li* et al.* and Gu* et al.* reported that berberine could inhibit intestinal epithelial tight junction damage caused by proinflammation cytokines [53]. Pretreatment with berberine could reduce the intestinal permeability and improve LPS-induced redistribution of tight junction-related protein claudin-1 and claudin-4 [54]. Combined, these results indicated that berberine improved bacterial endotoxin induced intestinal barrier disruption and play a significant role in the maintenance of the intestinal epithelial tight junction.

Besides berberine, other natural products also showed powerful functions to protect the intestine from LPS-induced gut microbiota. In a previous study the effects of curcumin were tested on Caco-2 cells and HT-29 cells, and it was found that curcumin could attenuate the disruption of intestinal epithelial barrier functions. Curcumin reduced the release of IL-1b secreted from LPS, induced IEC and macrophages, and prevented the disintegration of tight junction proteins, such as ZO-1, claudin-1, claudin-7, and actin filaments [55]. Therefore, curcumin can be a potential compound for treating intestine barrier injury through increasing the expression of tight junction proteins.

Similarly,* Flos Lonicera*, one of the most well-known traditional Chinese medicines, could modulate tight junctions at the cell-based level. It not only restored the side effects induced by LPS but also increased several microbiota, which had beneficial effects on maintaining the integrity of the intestinal barrier [18]. For example, Chelakkot* et al*. demonstrated that* akkermansia-muciniphila* could activate tight junction-related signaling of AMPK, especially in obesity and T2D patients [56]. In another study, it was suggested that the polyphenolic compound resveratrol (Res), which is found in grape seeds, grape skin, and red wine, attenuated intestinal barrier impairment and bacterial translocation induced by deoxynivalenol (DON) [57, 58]. Ling* et al*. reported that Res mainly facilitated claudin-4 expression to build up the tight junction complex and to resist DON-induced barrier dysfunction [59].

Together, these findings demonstrated that natural products can exert positive effects on the intestinal barrier by upregulating tight junction proteins, reducing inflammation, and increasing the abundance of probiotics. Gut microbiota disorders increased the secretion of LPS, thereby leading to a series of metabolic diseases [60, 61]. However natural products can directly or indirectly act on microbes to alleviate the condition.

## 5. Effect of Natural Products on Intestinal Mucosal Immunity

The intestinal mucosal immunity system consists of gut-associated lymphoid tissue (GALT), lymphocytes, and various immune-related factors. At present, it is believed that digestive system diseases, autoimmune diseases, pediatric allergic diseases, and tumors are closely related to intestinal mucosal immunity [62, 63]. Natural products may provide an alternative treatment approach for these diseases.

Many metabolic diseases are related to intestinal inflammation and gut microbiota, and it is the intestinal mucosal immunity that is the connection between them. As previously reported, the induction of IL-22 in innate lymphoid cells and CD4^+^T cells is impaired in obese mice, and IL-22 deficiency in mice has shown that mice are prone to developing metabolic disorders [64]. Intestinal mucosal immunity in obese mice contributed to obesity-related insulin resistance via preserving the gut barrier and remitting fat inflammation [65].

Gut microbiota regulates host innate and adaptive immunity. Dysbiosis of gut microbiota can cause severe intestinal diseases [66]. Segmented filamentous bacteria can activate CD4^+^ T cells and produce IL-17A to promote inflammation in the intestine [67]. Microbiota such as* Clostridium clusters* promoted the development of intestinal regulatory T cells (T reg), which are significantly decreased in colonic germ-free mice [68, 69]. Moreover, in the small intestine, TH17 cells secrete cytokines, such as IL-17/IL-22 to regulate the inflammation status in colon. However,* Clostridium arthromitus* and metabolites from microbiota, such as luminal adenosine triphosphate and tryptophan, promoted the development of TH17 cells [70–72]. Commensal bacteria modulated the abundance and activation of *γδ* T cells, which produced IL-17 to promote inflammation in the intestine [73]. Gut microbiota could also inhibit natural killer T cells (iNKT) [74, 75].

Innate lymphoid cells (ILCs) share functional characteristics with T cells in the lamina propria. Gut microbiota may regulate the ILC either through direct recognition or by indirect induction of cytokine secretion by other cells [76]. On the contrary, microbiota can also regulate the activation of ILCs via inducing the production of IL-25, which confirms the opinion that microbiota is important for homeostasis [77, 78].

IgA constitutes approximately 75% of the total antibody production in mammals and is the most abundant immunoglobulin in mucosal secretions [79]. Gut microbiota is a strong inducer of IgA production. For example, orally administered flagellin abrogated IgA secretion in the intestine [80].

Recent studies have shown that berberine had beneficial effects on intestinal immune cells and immune factors. Moreover, berberine also inhibited the expression of various immune factors and reduced the low-grade inflammation [81]. For example, berberine has been widely used in the treatment of UC via modulating T reg cells and TH cells in the colon [82]. Researchers in China revealed that berberine improved lipid metabolism in the liver by changing microbiota and by regulating bile acid metabolism and the FXR pathway in the intestine [83]. In addition, M. charantia improved insulin resistance via decreasing the F/B ratio in the intestines of diabetic rats [84]. Therefore, it improved the inflammation status in the intestine and diabetes mellitus [85]. Curcumin could improve pancreatic *β* cells and decrease glucose levels, as well as other metabolic profile in T2D or atherosclerosis through inhibition of iNOS and COX-2 [86, 87]. Furthermore, a curcumin-supplemented diet increased the richness of* lactobacillales* and improved the index of colon tumors [88]. Ginsenoside also protected cardiac function and decreased blood glucose levels [89].

Natural products modulate the immune status via changing the level of immune factors, such as IL-22, as well as activating T reg cells or inhibiting the development of Th17 cells. Natural products can also decrease systemic inflammation via improving insulin resistance as well as other metabolic profiles. Natural products have promising therapeutic effects on metabolic diseases by improving the immune status in the body.

## 6. Conclusion

In the past, numerous studies have demonstrated that natural products derived from traditional Chinese medicine implement their pharmacological properties through restoring gut homeostasis, including alteration of microbiota composition, adjustment of microbiota metabolites, enhancement of the expression of tight junction proteins, and enhancing mucosal immunity. Therefore, we conclude that gut microbiota and its subsequent changes of intestinal environment play very important roles in mediating the pharmacological effects of natural products. Given the fact that various roles of gut microbiota were gradually elucidated in human diseases and health, we hypothesize that the important roles of gut microbiota will increase and will be more realized and illuminated. It is to be expected that an increased number of potential drug targets arising from gut microbiota will be discovered.
